# The Perks of Doing Housework: Its Impacts on Physical Health, Mental Well-Being, Cognitive Performance, and Survival

**DOI:** 10.1093/geroni/igab046.414

**Published:** 2021-12-17

**Authors:** Li Chu, Xianmin Gong, Jennifer Lay, Fan Zhang, Timothy Kwok, Helene Fung

**Affiliations:** 1 Stanford University, Stanford University, California, United States; 2 The Chinese University of Hong Kong, Hong Kong, Hong Kong; 3 University of Exeter, Exeter, England, United Kingdom; 4 Jinan University, Jinan University, Guangzhou, Guangdong, China (People's Republic); 5 The Chinese University of Hong Kong, Shatin, N.T., Hong Kong, Hong Kong

## Abstract

Previous research has shown mixed results regarding the effects of doing housework. While some earlier studies have found no association between performing heavy housework and health, other studies have found various benefits of doing housework, including body leanness and lower mortality rate. This study examined the effects of housework on older adults’ survival over a period of 14 years, and investigated the underlying mechanisms. A total of 2,768 older adults in Hong Kong (female: 47.29%; age: 65-98) from a longitudinal survey study were included in the current analyses. Linear regression analysis revealed that doing more housework was significantly associated with surviving more days (β = 45.36, SE = 6.40, p < .001). We then examined whether the association between housework and survival was mediated by physical health, mental health and/or cognitive functioning using a parallel mediation model with multiple mediators. Results showed a significant partial mediating effect of physical health (β = 1.20, SE = .53, p = .003), a marginally significant partial mediating effect of cognitive functioning (β = 1.35, SE = .70, p = .054) and no mediating effect of mental health. All the analyses remained consistent after controlling for sex, education, marital status, subjective social status and living arrangement. These results suggest that doing housework may benefit survival by improving physical and cognitive functioning. Our findings have implications for better understanding factors that influence mortality, developing accessible physical activity interventions for older adults, and supporting aging in place.

